# Frühe Hilfen aus der Distanz – Chancen und Herausforderungen bei der Unterstützung psychosozial belasteter Familien in der COVID-19-Pandemie

**DOI:** 10.1007/s00103-021-03450-6

**Published:** 2021-11-03

**Authors:** Ilona Renner, Juliane van Staa, Anna Neumann, Frank Sinß, Mechthild Paul

**Affiliations:** Nationales Zentrum Frühe Hilfen, Bundeszentrale für gesundheitliche Aufklärung, Maarweg 149–161, 50825 Köln, Deutschland

**Keywords:** Frühe Hilfen, Pandemie, Versorgungskonzept, Qualitätsentwicklung, Fachkräfte, Vulnerable Familien, Early childhood intervention, Pandemic, Care concept, Quality development, Health professionals, Vulnerable families

## Abstract

Kontaktbeschränkende Maßnahmen waren zur Eindämmung des SARS-CoV-2-Infektionsgeschehens ab Frühjahr 2020 in Deutschland notwendig. Jedoch stellten sie Familien, insbesondere Familien in Belastungslagen, vor besondere Herausforderungen. Der Beitrag geht der Frage nach, inwieweit sich die Coronapandemie bis zum Mai 2021 auf die Lebenssituation der Familien ausgewirkt hat und wie eine längerfristige Betreuung im Kontext der Frühen Hilfen fortgeführt werden konnte. Die Analysen basieren hauptsächlich auf einer qualitativen Studie mit psychosozial belasteten Müttern von jungen Kindern, 2 Befragungen von Gesundheitsfachkräften, die Familien in den Frühen Hilfen längerfristig unterstützen, sowie einer Befragung von kommunalen Akteuren, die für die Steuerung und Umsetzung der Frühen Hilfen in den Kommunen verantwortlich sind.

Psychosozial belastete Familien erleben existenzielle Ängste und eine generelle Überforderung in der Pandemie. Innerfamiliale Konflikte scheinen zuzunehmen. Die längerfristige Unterstützung durch die Frühen Hilfen wurde vom persönlichen Kontakt im häuslichen Umfeld vorrangig in die Distanz verlagert. Obwohl im Verlauf der Pandemie viele Fachkräfte wieder zum ursprünglichen Versorgungskonzept zurückkehrten, werden Elemente der „Hilfe auf Distanz“ weiterhin ergänzend eingesetzt.

Aufgrund der pandemiebedingten zusätzlichen Belastungen der Familien, die in den Frühen Hilfen begleitet werden, war die Fortführung der Unterstützung noch wichtiger als ohnehin schon. Die Formate einer „Hilfe auf Distanz“, die in der Pandemie notgedrungen erprobt wurden, könnten das Potenzial haben, die Hilfeleistungen der Gesundheitsfachkräfte in den Frühen Hilfen zu ergänzen und so zu einem Qualitätsentwicklungsschub beizutragen.

## Abstract (en)

As of spring 2020, contact-restricting measures were necessary to contain the incidence of infection with the SARS-CoV‑2 virus in Germany. However, these restrictions posed particular challenges to families, especially families in high-stress situations. This paper examines how the COVID-19 pandemic affected the life of families and if long-term support in the context of early childhood intervention could be continued. The following analyses are based on qualitative research with psychosocially stressed mothers of young children, two surveys of health professionals who provide long-term support to families in early childhood intervention, and a survey of municipal actors who are responsible for the management and implementation of early childhood intervention in German communities.

During the pandemic, families in high-stress conditions report existential fears and general overstrain. Intrafamilial conflicts seem to increase. Home visits in the context of long-term psychosocial support were initially primarily replaced by phone calls, video calls or other forms of “support from a distance.” During the course of the pandemic, many professionals have begun conducting home visiting again, but several elements of support from a distance continue to be used as a supplement.

Due to the pandemic-related additional burdens on the families who were accompanied by early childhood intervention, the continuation of support was even more important than it already was. Having been tested by necessity during the pandemic, several formats of support from a distance may have the potential to complement the usual assistance provided by health professionals in early childhood intervention and thus contribute to its quality development push.

## Einleitung

**Figure 1:**
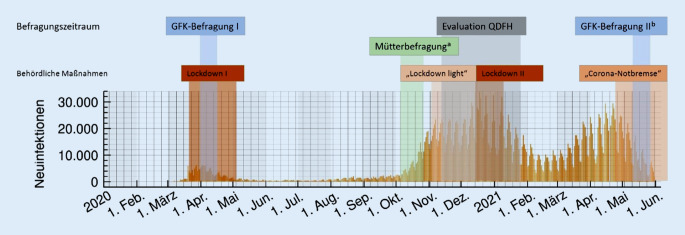

Zur Eindämmung der COVID-19-Pandemie wurden in den Jahren 2020 und 2021 kontaktbeschränkende Maßnahmen behördlich angeordnet (Abb. 1). Diese Maßnahmen waren aus epidemiologischer Sicht notwendig, aber mit stark erhöhten Belastungen für Familien verbunden.

Insbesondere Eltern von jüngeren Kindern empfanden während des ersten Lockdowns ihre Situation als belastend [1]. Die allgemeine Lebenszufriedenheit nahm ab [2]. Zudem wurden Partnerschaftskonflikte während des ersten Lockdowns von Eltern jüngerer Kinder deutlich häufiger berichtet als von Müttern und Vätern älterer Kinder oder kinderlosen Paaren [1, 3]. Diese belasteten Familiensituationen wirkten sich nachteilig auf die psychische Gesundheit von Kindern aus [4, 5].

Noch deutlicher als im repräsentativen Familienquerschnitt hat sich die Situation für psychosozial belastete Familien verändert: Familien, die ohnehin mit besonderen Herausforderungen leben, wie bspw. Armut oder psychischer Erkrankung, mussten – zusätzlich zur Bewältigung dieser grundsätzlichen Herausforderungen – die pandemiebedingten Belastungen kompensieren [6, 7]. Gleichzeitig konnten aufgrund der Maßnahmen zum Infektionsschutz spezifische Unterstützungsleistungen wie die Frühen Hilfen (Infobox 1), die häufig auf direkten persönlichen Kontakten beruhen, nicht oder nicht wie gewohnt eingeleitet und weitergeführt werden.

Das Nationale Zentrum Frühe Hilfen (NZFH) hat, gefördert aus Mitteln der Bundesstiftung Frühe Hilfen des Bundesministeriums für Familie, Senioren, Frauen und Jugend, frühzeitig und ad hoc pandemiebegleitende, empirische Untersuchungen durchgeführt (Infobox 2; Abb. 1). Dies war notwendig, um der Situation angepasste Entwicklungen anzustoßen [8].

Da erste Erkenntnisse sehr zeitnah generiert werden mussten und psychosozial belastete junge Eltern in Bevölkerungsstichproben stark unterrepräsentiert waren, wurden alternative forschungspragmatische Zugänge gewählt: zum einen Interviews und Fokusgruppen mit kleinen Stichproben psychosozial belasteter Mütter, zum anderen Onlineerhebungen bei Gelegenheitsstichproben mit Fachkräften in den Frühen Hilfen. So konnten erste Eindrücke aus verschiedenen Blickwinkeln gewonnen werden.

Im Folgenden beleuchten wir anhand dieser Quoten- bzw. Gelegenheitsstichproben zunächst die Situation und Versorgung der Familien im Frühjahr 2020, dann skizzieren wir Entwicklungen bis zum Frühjahr 2021. Da sich die Digitalisierung bzw. die „Hilfe auf Distanz“ als zentrales Entwicklungsthema der Frühen Hilfen herauskristallisiert, fragen wir anschließend, inwieweit die Erfahrungen aus der Coronapandemie Optionen für einen Qualitätsentwicklungsschub in den Frühen Hilfen bieten.

### Infobox 1 Frühe Hilfen

Frühe Hilfen sind Angebote für Eltern ab der Schwangerschaft und Familien mit Kindern bis 3 Jahre. Sie bieten Eltern Unterstützung, Beratung und Begleitung. Sie sind freiwillig und kostenfrei. Ziel ist es, jedem Kind eine gesunde Entwicklung und ein gewaltfreies Aufwachsen zu ermöglichen.

Gesundheitsfachkräfte in den Frühen Hilfen sind Familienhebammen und Familien‑, Gesundheits- und Kinderkrankenpflegerinnen, die Familien niedrigschwellig und aufsuchend betreuen und begleiten. Ihre Aufgaben umfassen beispielsweise die alltagspraktische Unterstützung von Familien und die Förderung von Kompetenzen der Eltern in der Versorgung und Erziehung ihrer Kinder.

Weitere Informationen finden Sie unter www.fruehehilfen.de und www.elternsein.info.

### Infobox 2 Empirische Studien des Nationalen Zentrums Frühe Hilfen (NZFH) seit Beginn der COVID-19-Pandemie

*Evaluation der Qualitätsdialoge Frühe Hilfen (QDFH)*.

Die „Qualitätsdialoge Frühe Hilfen“ sollen die Frühen Hilfen qualitätsgesichert weiterentwickeln. Von 2018 bis 2021 nehmen 23 Kommunen an diesem Projekt teil. Dieser Praxisprozess wird wissenschaftlich begleitet. Die hier vorgestellten Ergebnisse basieren auf Daten der 2. Erhebungswelle der quantitativen Onlinebefragung und auf qualitativen, leitfadengestützten Befragungen zwischen Januar und März 2021. An der Onlinebefragung nahmen 302 Akteure der beteiligten Kommunen teil. Leitfadeninterviews wurden mit 12 Steuerungsverantwortlichen der strategischen und operativen Ebene geführt. Bezogen auf die COVID-19-Pandemie wurden 9 Items in den quantitativen Onlinefragebogen integriert und der Interviewleitfaden um das Thema „Arbeiten unter Coronabedingungen“ ergänzt.

*Gesundheitsfachkräftebefragung I (Frühjahr 2020)*.

Bei der Gesundheitsfachkräftebefragung I [10] handelt es sich um eine qualitative Ad-hoc-Befragung von Familienhebammen und Familien‑, Gesundheits- und Kinderkrankenpflegenden. Die Rekrutierung der Teilnehmenden erfolgte im Schneeballverfahren, der Befragungslink wurde initial an *n* = 5 Fachkräfte versandt, die diesen weiterleiteten. Der Onlinefragebogen enthielt überwiegend offene Fragen zur beruflichen Situation der Fachkraft und zur Situation in den betreuten Familien. Die Feldzeit dauerte vom 30.03. bis zum 14.04.2020. Der Datensatz umfasst Antworten von *n* = 58 Gesundheitsfachkräften, die in der längerfristigen aufsuchenden Betreuung tätig sind.

*Gesundheitsfachkräftebefragung II (Frühjahr 2021)*.

Im Mai 2021 (Feldzeit 11.–26.05.) wurden erneut Fachkräfte befragt, um ein aktuelles Bild der Lage in den Frühen Hilfen zu erhalten. Die Rekrutierung erfolgte wieder im Schneeballsystem mit einem initialen Versand an *n* = 43 Fachkräfte. Der Fragebogen enthielt hauptsächlich geschlossene Fragen, entwickelt auf Basis der Ergebnisse der ersten Fachkräftebefragung. Insgesamt nahmen *n* = 82 Fachkräfte an der Befragung teil.

*Befragung von Müttern zu ihrer Situation im ersten Lockdown*.

Um auch die Sichtweise der Familien selber berücksichtigen zu können, wurden im November 2020 3 Minifokusgruppen mit je 3 Müttern und 6 Einzelinterviews mit 5 weiteren Müttern durchgeführt. Die Teilnehmenden wurden so ausgewählt, dass neben dem Merkmal „niedrig gebildet“ mindestens ein weiteres Belastungsmerkmal vorlag. Retrospektiv wurde das Alltagsleben der teilnehmenden Mütter während der Zeit der kontaktbeschränkenden Maßnahmen im Frühjahr 2020 exploriert.

## Situation von psychosozial belasteten Familien (in den Frühen Hilfen)

Die empirische Forschung zur Situation von Familien in Belastungslagen identifizierte folgende zentrale Themenbereiche: existenzielle Nöte der Familien, Überforderung und eskalierende Konflikte.

### Existenzielle Nöte

Eine Repräsentativbefragung des wirtschafts- und sozialwissenschaftlichen Instituts der Hans-Böckler-Stiftung (WSI) zeigt, dass pandemiebedingte „große Sorgen um die eigene wirtschaftliche Situation“ und eine „äußerst oder stark belastende finanzielle Situation“ umso stärker ausgeprägt waren, je niedriger das Haushaltseinkommen war [9]. Dabei ist nicht nur die Linearität dieses Zusammenhangs, sondern auch das Ausmaß der Unterschiede bemerkenswert: Während im April 2020 7–14 % der Befragten in den 3 höchsten Einkommensgruppen angaben, besorgt um ihre eigene wirtschaftliche Situation zu sein, lag dieser Anteil in den 3 niedrigsten Einkommensgruppen mit 30–41 % deutlich höher [9].

Übereinstimmend mit den Ergebnissen dieser Repräsentativbefragung berichteten auch die Gesundheitsfachkräfte, die Familien in den Frühen Hilfen betreuen und begleiten, dass sie bei den Familien zusätzliche finanzielle und existenzielle Belastungen wahrnehmen [10]: Familien hatten – ausgelöst durch den ersten Lockdown im Frühjahr 2020 – „existenzielle Ängste“, „Sorgen um die finanzielle Absicherung des Lebensunterhalts“ und Angst um den Verlust des Arbeitsplatzes. Zusätzlich erschwerten geschlossene Ämter und dadurch Verzögerungen bei Antragstellung bzw. -bearbeitung die finanzielle Lage der Familien.

Existenzielle Ängste wurden auch in der qualitativen Befragung von 14 Müttern in Belastungslagen, die das NZFH im November 2020 durchführte (vgl. Infobox 2, Abschnitt 4), von den Befragten zentral thematisiert. Rückblickend erklärte eine der befragten Mütter exemplarisch für den Diskussionsverlauf in den Fokusgruppen, dass es „mit der Arbeit sehr schwierig“ sei. „Man hat darum Angst, auch den Job zu verlieren.“ Dabei führte das Bewusstsein, als Eltern Verantwortung für das (materielle) Wohl der eigenen Kinder zu tragen, zu besonderem Druck:

Gut, wäre man alleine ohne Kinder, wäre es nicht so schlimm, das Finanzielle. Aber wenn man schon Kinder hat und wenn man weiß, was ein Kind kostet, da hat man echt Angst irgendwie, dass es doch am Geld fehlen könnte.

### Überforderung

Neben den materiellen Sorgen belastete die Familien auch die veränderte Alltagssituation. Die Überforderung war – aus Sicht der Gesundheitsfachkräfte – insbesondere darauf zurückzuführen, dass die ganze Familie inklusive der Kinder permanent zu Hause war, dass die Kinderbetreuung bzw. andere Unterstützungsleistungen wegbrachen und der Alltag von den Familien alleine bewältigt werden musste [10].

Den veränderten Alltagsroutinen konnte ein Teil der Familien in Deutschland auch positive Seiten abgewinnen, im Sinne von „Entschleunigung“ und „weniger Freizeitstress“ [11]. Vereinzelt berichteten auch die Mütter in der NZFH-Stichprobe von positiven Aspekten der kontaktbeschränkenden Maßnahmen, etwa dass sie die Möglichkeit boten, „mein Kind besser kennengelernt“ zu haben. Weit überwiegend wurde jedoch dargestellt, wie die Anforderungen in der Pandemie die Bewältigungsmöglichkeiten der Familien zu übersteigen drohten, insbesondere mit Blick auf die alleinige Verantwortung für Betreuung, Bildung und Unterhaltung der Kinder rund um die Uhr und oft auf engstem Raum.

Frühmorgens, wenn die Augen aufgehen: „Mama!“, bis spätabends, wenn die Augen zugehen: „Mama!“. Alle zwei Sekunden Mama hier, Mama da.

In Anbetracht der beengten Wohnverhältnisse, die von Müttern in Armutslagen geschildert wurden („Wir haben eine 3‑Zimmer Wohnung in einem Hochhaus“), erscheint es durchaus plausibel, dass ihnen vielfach „die Decke auf den Kopf“ fiel. Daraus resultierten akute Stressreaktionen, die von den Müttern als „nicht ganz gesund“ wahrgenommen wurden, bis hin zu Anzeichen für eine beginnende psychische Symptomatik, die von einer Mutter geschildert wurde:

Da hat man so depressive Phasen, denkt man zu viel nach, kommt man auf komische Ideen dann und so.

### Eskalierende Konflikte

Mehr als die Hälfte der Gesundheitsfachkräfte gab 2020 in der ersten Fachkräftebefragung des NZFH an, dass die Situation im ersten Lockdown das Risiko für Gewalt in den Familien erhöht hat: Sie befürchteten, dass schwelende Konflikte verstärkt werden bzw. eskalieren könnten [10]. Diese Einschätzung wurde von Kinderschutzexpertinnen und -experten in Deutschland sowie der medialen Öffentlichkeit geteilt [12]. Auch die kommunalen Akteure, die vor Ort für die Umsetzung der Frühen Hilfen verantwortlich sind, stimmten Ende 2020 und Anfang 2021 in einer NZFH-Befragung (Infobox 2) zu 70 % der Aussage: „Die Situation in vielen Familien macht mir große Sorgen“, zu.

Die Frage, inwieweit sich die Befürchtung einer Zunahme von Gewalt in Familien bestätigt hat, ist nicht endgültig geklärt. Im Jugendhilfebarometer, einer regelmäßigen Befragung des Deutschen Jugendinstituts, wurden für den (Lockdown‑)Zeitraum vom 23.04. bis zum 12.05.2020 *keine* erhöhten Fallzahlen bei den Gefährdungseinschätzungen gem. § 8a Achtem Buch Sozialgesetzbuch (SGB VIII) – Schutzauftrag bei Kindeswohlgefährdung – festgestellt [13]. Für den medizinischen Kinderschutz konnte im März/April 2020 bei einem Vergleich der Anzahl von Kinderschutzfällen in der stationären und ambulanten Versorgung mit den Fallzahlen von 2019 (März/April) sogar ein Rückgang um 20 % bzw. 15 % beobachtet werden [14].

In eine andere Richtung weisen Zahlen, die die längerfristige Entwicklung bis Ende 2020 abbilden. In der „8a-Zusatzerhebung“ der Arbeitsstelle Kinder- und Jugendhilfestatistik, die seit Mai 2020 fortlaufend die abgeschlossenen Gefährdungseinschätzungen durch die Jugendämter erfasst, wurden insbesondere im Juni sowie im Herbst (ab September) überproportional viele 8a-Verfahren gemeldet, was „wahrscheinlich auf Auswirkungen der Pandemie zurückzuführen“ sei [15]. Die gehäuften Fälle ab Herbst können zwar einerseits mit „Nachholeffekten“ verspätet bemerkter und übermittelter Fälle zu tun haben, andererseits könnten sich auch die familiären Belastungen mit dem längeren Andauern der Pandemie verstärkt und zu vermehrten Kindeswohlgefährdungen geführt haben [15]. Auch die im Juli veröffentlichten Zahlen des Statistischen Bundesamtes belegen, dass die Jugendämter im Vergleich zu 2019 für das Gesamtjahr 2020 ca. 9 % *mehr* Kindeswohlgefährdungen festgestellt haben (Höchststand seit Beginn der Erhebungen in 2012). Damit setzte sich ein Trend ansteigender Fallzahlen weiter fort, der Anstieg für 2020 bewegte sich in einer vergleichbaren Größenordnung wie der Anstieg in den 2 Jahren vor der Pandemie [15]. Dass sich die Fallzahlen im Jahr 2020 – trotz der pandemiebedingten höheren Belastungen der Familien – nicht stärker erhöht haben, wird auch im Zusammenhang eines möglicherweise gestiegenen „Dunkelfeldes“ diskutiert: Der Anteil nicht entdeckter Kindeswohlgefährdungen könnte während der Pandemie aufgrund des verstärkten Rückzugs ins Private sowie reduzierter sozialer Kontrollen und eingeschränkter Meldewege stärker angewachsen sein [13–16].

## Psychosoziale Versorgung durch Frühe Hilfen

### Vom Hausbesuch zur „Hilfe auf Distanz“

Die kontaktbeschränkenden Maßnahmen während des Lockdowns im Frühjahr 2020 wirkten sich nicht nur auf die Situation der Familien, sondern auch auf die längerfristige Betreuung in den Frühen Hilfen aus, da diese Hilfeleistung in ihrer Grundkonzeption auf persönlichen Kontakten im häuslichen Umfeld der betreuten Familien beruht: Viele Fachkräfte hatten – in Reaktion auf die Anforderungen des Infektionsschutzes – ihre Beratungs- und Betreuungstätigkeit ausschließlich oder überwiegend vom Hausbesuch auf die telefonische Begleitung verlagert, auch per Video, Skype, Kurznachricht oder E‑Mail fanden Beratungen statt [10]. Hausbesuche wurden häufig nur noch bei sehr stark belasteten Familien oder zur Krisenintervention durchgeführt, unter Beachtung der Abstands- und Hygieneregelungen sowie teilweise auch im Freien oder mit kürzerer Dauer [6].

### Beurteilung der Veränderungen und Maßnahmen

Die Fachkräfte betonten zwar die Notwendigkeit der kontaktbeschränkenden Maßnahmen, es überwogen jedoch Angaben, die auf die damit verbundenen Herausforderungen hinwiesen [10]: Jede vierte Fachkraft beschrieb ihre Arbeit im Lockdown als „erschwert“, „belastend“ oder „nicht zufriedenstellend“. Mit Sorge wurde auch auf die Qualität der Betreuungsarbeit geblickt, der persönliche Kontakt und Eindruck fehlten, was mit der Befürchtung einherging, in den Familien „etwas zu übersehen“.

Ähnlich zwiegespalten war auch die Sicht der Mütter. Einerseits wurden die kontaktbeschränkenden Maßnahmen zur Eindämmung des Infektionsgeschehens während des ersten Lockdowns angenommen und verstanden. Andererseits bestätigten diejenigen Mütter, die zur Zeit des Lockdowns in der längerfristigen Familienbegleitung der Frühen Hilfen waren (*n* = 2), die Aussagen der Fachkräfte, wonach die Verlagerung der Familienunterstützung vom persönlichen Kontakt in die Distanz zu qualitativen Einbußen der Hilfeleistung führt: Obwohl die telefonische oder digitale Betreuung „auch geklappt“ habe, fehlte die persönliche, Sicherheit gebende Ebene.

## Situation und Versorgung der Familien in den Frühen Hilfen im zweiten Pandemiejahr

Wie geht es den Familien nach einem Jahr in der Pandemie und inwieweit hat sich die psychosoziale Versorgung den pandemischen Bedingungen angepasst? Um auf diese Fragen eine Antwort zu finden, hat das NZFH im Mai 2021 nochmals Gesundheitsfachkräfte in der längerfristigen, aufsuchenden Betreuung und Begleitung der Frühen Hilfen gebeten, ihre Expertise und Erfahrungen mit dem NZFH zu teilen.

### Die Situation der Familien

Nach einem Jahr unter Pandemiebedingungen war die Situation der betreuten Familien aus Sicht der im Frühjahr 2021 befragten Fachkräfte weiterhin angespannt. Wie auch in der Befragung ein Jahr zuvor sah über die Hälfte der Fachkräfte unverändert „finanzielle Nöte“, „existenzielle Ängste“ oder „wirtschaftliche Engpässe“ in „(fast) allen“ oder „vielen“ der von ihnen betreuten Familien. Knapp die Hälfte der zweiten Gesundheitsfachkräftestichprobe beobachtete ein im Vergleich zu Zeiten vor der Pandemie erhöhtes Konfliktniveau zwischen den Eltern, 20 % ein erhöhtes Risiko für Partnergewalt und 31 % für Kindeswohlgefährdungen. Sehr stark ausgeprägt war aus Sicht der Fachkräfte auch im Frühjahr 2021 die „Überforderung mit der Situation, dass die Kinder viel zuhause sind“ (75 %), häufig erschwert durch wenig Rückzugsmöglichkeiten in beengten Wohnverhältnissen (68 %).

Nach einem Jahr mit wiederkehrenden Lockdowns wurde von den 2021 befragten Fachkräften wahrgenommen, dass die von ihnen betreuten Familien aufgrund immer noch fehlender Unterstützungsangebote weiterhin auf sich alleine gestellt waren (87 %). Knapp 80 % der Fachkräfte berichteten von einer sozialen Vereinsamung der Eltern. Ähnlich hoch war der Anteil der Fachkräfte, der eine soziale Vereinsamung bei den Kindern wahrnahm. Zudem vermuteten die befragten Familienhebammen und Familien‑, Gesundheits- und Kinderkrankenpflegenden „in (fast) allen“ oder „in vielen“ der betreuten Familien Nachteile für die Kinder durch reduzierte Förderung (82 %). Zudem befürchteten sie die Entstehung oder Verstärkung von Entwicklungs- oder Verhaltensauffälligkeiten bei den Kindern (48 %) sowie psychische Probleme bzw. Erkrankungen der Eltern (55 %).

### Psychosoziale Versorgung der Familien

Die zweite Fachkräftebefragung des NZFH deutet auf eine gegenüber dem ersten Lockdown wieder etwas normalisierte Umsetzung der (aufsuchenden) längerfristigen Betreuung und Begleitung im Kontext der Frühen Hilfen hin, ohne dass jedoch das „Vor-Pandemie-Niveau“ wieder erreicht wäre.

Knapp 60 % der befragten Fachkräfte gaben im Mai 2021 an, dass sich die Anzahl der durchgeführten Hausbesuche gegenüber dem ersten Lockdown vor einem Jahr wieder „etwas“ oder „deutlich“ erhöht habe, bei einem Viertel gab es gegenüber dem ersten Lockdown vor einem Jahr hingegen keine Veränderung. 17 % berichteten sogar von einer aktuell noch weiter reduzierten Anzahl von Hausbesuchen.

Obwohl der persönliche Kontakt zu den Familien nach Aussage der im Mai 2021 befragten Fachkräfte insgesamt wieder häufiger stattfand, blieben telefonische und neue digitale Lösungen, die alternativ oder ergänzend zum Hausbesuch eingesetzt wurden, weiterhin von Bedeutung. Die Begleitung der Familien erfolgte bis zum Erhebungszeitpunkt im Mai 2021 weiterhin ergänzend zu den verstärkt wieder aufgenommenen persönlichen Kontakten insbesondere telefonisch und per Messengerdienste: Jeweils ca. 2 Drittel der Fachkräfte gaben an, dass sie diese Medien in den letzten 3 Monaten „immer/häufig“ oder „manchmal“ eingesetzt haben. Auch Beratungen mit Bildübertragung (Videochat, Skype etc.) wurden durchgeführt: Hier gab rund jede vierte Fachkraft an, diese Art der Beratung „immer/häufig“ oder „manchmal“ zu nutzen.

Die Ergebnisse zeigen ein stark heterogenes Bild der Versorgung psychosozial belasteter Familien: Trotz eines allgemeinen Trends der Rückkehr zum ursprünglichen Konzept, scheinen im zweiten Jahr der Pandemie sehr unterschiedliche Modelle der längerfristigen Betreuung und Begleitung von Familien zu koexistieren.

## „Hilfe auf Distanz“: Notnagel oder Chance?

Telefonische sowie neue digitale Formate sind die zentralen Medien der „Hilfe auf Distanz“. Inwieweit hat der verstärkte Einsatz dieser Medien, der zunächst aus der Not des Infektionsschutzes heraus entstanden ist, einen Qualitätsentwicklungsimpuls in den Frühen Hilfen ausgelöst?

### Digitalisierung in der längerfristigen Betreuung und Begleitung durch Gesundheitsfachkräfte in den Frühen Hilfen

Im Mai 2021 berichtete etwa jede zweite Fachkraft (Top-2-Werte auf einer Antwortskala von 1 „trifft überhaupt nicht zu“ bis 5 „trifft voll und ganz zu“) von gestiegenen Kompetenzen bei der telefonischen bzw. digitalen Familienbegleitung. Der Kompetenzzuwachs erfolgte insbesondere aufgrund der Erfahrungen, die Fachkräfte mit diesen Formaten im Pandemiejahr sammeln konnten. Ebenfalls rund 45 % der Befragten gaben an, dass neue Softwarelösungen oder Apps erprobt bzw. diese Tools zur Nutzung zugelassen wurden. Allerdings hatte es nur wenig Unterstützung „von außen“ gegeben, um die „Hilfe auf Distanz“ erfolgreich umsetzen zu können: Nur jeweils ein Viertel der Fachkräfte gab an, eine bessere technische Ausstattung oder Fortbildungen zu digitalen Formaten erhalten zu haben. Dies deutet auf einen hohen Bedarf an Qualifizierungsangeboten hin. Auch bezüglich der Datenschutzthematik scheint sich nicht ausreichend viel bewegt zu haben: Nur gut jede dritte Fachkraft gab an, dass für die telefonische bzw. digitale Betreuung relevante Datenschutzaspekte geklärt wurden.

Obwohl telefonische und digitale Formate eine Zeitersparnis durch reduzierte Anfahrtswege sowie regelmäßigere Kontakte zu den Familien ermöglichten, was als Pluspunkt gewertet wurde, überwogen in der Wahrnehmung der Fachkräfte die Nachteile der „Hilfe auf Distanz“. Dennoch konnten sich 42 % der Fachkräfte vorstellen, dass Elemente der digitalen Betreuung über die Pandemie hinaus erhalten bleiben, und denken dabei vor allem an die Videotelefonie sowie generell an Telefonate oder Kurznachrichten, die für kurzfristige oder „kleinere“ Fragen oder Anliegen „zwischendurch“ geeignet erscheinen ebenso wie für das schnelle Reagieren in Akutsituationen. Außerdem relevant für „Nach-Pandemie-Zeiten“ könnten aus Sicht einiger Fachkräfte „Onlineelternabende“ bzw. digitale Vorträge zu speziellen Themen wie Schlafen oder Ernährung sein.

### Digitalisierung in der Umsetzung der Frühen Hilfen in den Kommunen

Wie auch in anderen Bereichen Deutschlands [16] hat die Coronapandemie Defizite in der Digitalisierung der kommunalen Verwaltung offengelegt. Beispiel dafür sind die 23 Kommunen, die an den Qualitätsdialogen Frühe Hilfen teilnahmen: Aufgrund der zeitlichen Koinzidenz von Evaluation und Krisenbewältigung konnte die wissenschaftliche Begleitforschung der Qualitätsdialoge Frühe Hilfen (siehe Infobox 2) die Bewältigung dieser Herausforderungen umfassend dokumentieren. Es zeigte sich, dass viele kommunale Akteure zu Beginn der Pandemie noch nicht die notwendige technische Ausstattung für Homeoffice hatten bzw. diese ihnen von den Kommunen nicht zur Verfügung gestellt wurde. Auch fehlten häufig anwendungsbezogenes Wissen und auf den digitalen Austausch zugeschnittene Datenschutzkonzepte.

Zur Wiedererlangung der Handlungsfähigkeit mussten diese Defizite schnell beseitigt werden. Dabei hat sich gezeigt, dass nicht alle Inhalte problemlos ins Digitale transferiert werden können: Digitale Formate betonen die formelle Seite, während der informelle Charakter von Vieraugengesprächen im Rahmen von Präsenzveranstaltungen im digitalen Raum nicht abgebildet werden kann. Dies wurde von mehreren befragten Netzwerkkoordinatorinnen und -koordinatoren kritisch angemerkt [17].

**Figure 2:**
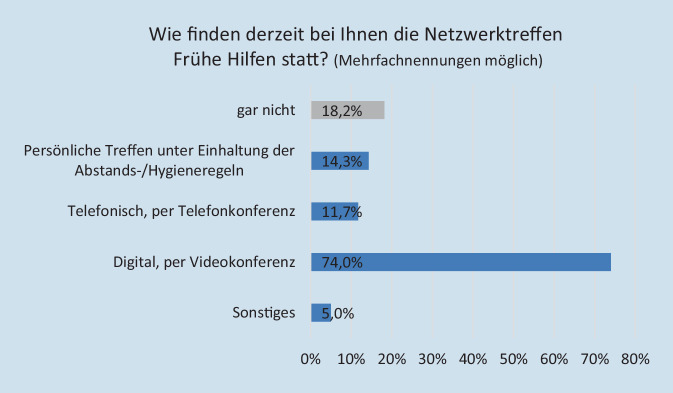

Im Mai 2021 fand laut den Angaben der Fachkräfte die Mehrzahl der Netzwerktreffen telefonisch oder per Videokonferenz statt, einige Treffen fielen ganz aus (Abb. 2). Es überwog eine kritische Einschätzung: Mit der digitalen Durchführung der Netzwerktreffen war nur rund jede dritte Fachkraft (sehr) zufrieden (Werte 4 und 5 auf einer fünfstufigen Skala von 1 „gar nicht zufrieden“ bis 5 „sehr zufrieden“). Bemängelt wurde insbesondere das Fehlen der persönlichen Ebene, es gingen „Nuancen im Gespräch verloren“, der „direkte Austausch“ komme zu kurz. Dennoch wurde auch von den Vorteilen digitaler Treffen berichtet, wie bspw. dem Wegfall von Fahrtwegen.

## Diskussion

Im vorliegenden Beitrag wurde gezeigt, dass die kontaktbeschränkenden Maßnahmen existenzielle Nöte und eine generelle Überforderungssituation in vielen psychosozial belasteten Familien auslösten. Gleichzeitig konnten die Frühen Hilfen zur Unterstützung der Familien nicht wie gewohnt eingeleitet oder fortgeführt werden. Statt im persönlichen Kontakt wurde die Hilfe teilweise und dauerhaft in die digitale Distanz verlagert. Gleichzeitig fanden auch die professionellen Kontakte zwischen den Fachkräften und Koordinierenden vermehrt im virtuellen Raum statt. Beides wird durchaus kritisch gesehen, birgt aber auch Chancen und wirft generell die Frage auf, inwieweit die Hilfe aus der Distanz eine Option für eine Weiterentwicklung der Frühen Hilfen sein kann.

Die im vorliegenden Beitrag dargestellten Analysen basieren hauptsächlich auf kleinen Stichproben psychosozial belasteter Mütter und Gelegenheitsstichproben von Gesundheitsfachkräften, die Familien in den Frühen Hilfen längerfristig begleiten, sowie von Akteuren der kommunalen Umsetzung und Steuerung. Die Ergebnisse haben sehr zeitnah belastbare Anhaltspunkte für die Situation und Versorgung von Familien in den Frühen Hilfen während der pandemiebedingten Kontaktbeschränkungen geliefert. Die Ergebnisse aus den 3 Fachkräftebefragungen können jedoch verzerrt sein, da die Stichproben aufgrund der Selbstselektion im angewandten Schneeballverfahren und der geringen Anzahl befragter Personen nicht repräsentativ sind. Zudem ist nicht auszuschließen, dass die Fachkräfte und möglicherweise auch die Mütter ein Interesse daran hatten, die Situation zu „dramatisieren“. Die Aussagen der Befragten weisen jedoch jeweils so deutlich in eine gemeinsame Richtung, dass ein Realitätsgehalt vermutet werden kann, ohne dabei einzelnen Prozentangaben zu viel Gewicht beizumessen. Bei der Interpretation der Angaben muss auch bedacht werden, dass Ergebnisse der Fachkräftebefragung zur Situation der Familien nicht auf diejenigen Familien in Belastungslagen zu verallgemeinern sind, die nicht von Fachkräften Früher Hilfen begleitet werden.

## Fazit

Familien in Belastungslagen, die von den Frühen Hilfen betreut werden, haben auch in der Coronapandemie wertvolle Unterstützung durch Gesundheitsfachkräfte erhalten, wenn auch häufig in veränderter Form und „auf Distanz“. Die Pandemie hat der Digitalisierung in den Frühen Hilfen Vorschub geleistet, sowohl in der Arbeit mit Familien als auch hinsichtlich des professionellen Austauschs.

Mithilfe digitaler Medien konnte der Kontakt zu den Familien vielfach aufrechterhalten werden. Die meisten Befragten – sowohl Familienbegleiterinnen als auch Mütter – schilderten jedoch ambivalente Erfahrungen mit der „Hilfe auf Distanz“: Während viele Inhalte gut transportiert werden konnten, fehlte u. a. die Vertrautheit des persönlichen Gesprächs. Als vorläufiges Fazit der pandemiebedingten Ausnahmesituation kann man festhalten, dass „Hilfe auf Distanz“ eine sinnvolle Ergänzung der bisherigen Beratungsformate ist, die spezifische Vorteile (u. a. kein Infektionsrisiko, Zeitersparnis, Spontanität) mit sich bringt, ohne eine persönliche Beratung jedoch vollständig ersetzen zu können. In der Weiterentwicklung der Unterstützungsleistung sollten auch in Zukunft, nach Abflauen der Pandemie, beide Formate berücksichtigt und bedarfsgerecht eingesetzt werden.

Dies gilt auch für den professionellen (und interprofessionellen) Austausch der Akteure, die Frühe Hilfen in den Kommunen umsetzen. Kooperation und Vernetzung sind das Herzstück der Frühen Hilfen. Hier mussten während der Lockdowns sehr schnell digitale Lösungen gefunden werden, um die Netzwerke in der Krisensituation weiterhin mit Leben zu füllen. Dabei zeigte sich, dass dies sogar Vorteile birgt. Gleichzeitig wurde den Beteiligten aufgrund eigener Erfahrungen aber bewusst, dass persönliche Kontakte auch zwischen Fachkräften unerlässlich sind.
